# IGF-I Medium Supplementation Improves Singly Cultured Cat Oocyte Maturation and Embryo Development In Vitro

**DOI:** 10.3390/ani11071909

**Published:** 2021-06-27

**Authors:** Lorena Fernandez-Gonzalez, Valeria Kozhevnikova, Eugeny Brusentsev, Stefanie Jänsch, Sergei Amstislavsky, Katarina Jewgenow

**Affiliations:** 1 Department of Reproduction Biology, Leibniz Institute for Zoo and Wildlife Research, Alfred-Kowalke-Straße 17, 10315 Berlin, Germany; jaensch@izw-berlin.de (S.J.); jewgenow@izw-berlin.de (K.J.); 2 Institute of Cytology and Genetics, Siberian Branch of the Russian Academy of Sciences, Prosp. Lavrent’eva 10, 630090 Novosibirsk, Russia; kozhevnikova.it@yandex.ru (V.K.); zerall@bk.ru (E.B.); amstis@yandex.ru (S.A.)

**Keywords:** IGF-I, IGF-II, GM-CSF, felids, cat oocyte, maturation, embryo culture, cat embryo, IVF, ICSI

## Abstract

**Simple Summary:**

Routine embryo production is the cornerstone for several research topics such as assisted reproduction techniques. For this, suitable culture media are needed to achieve satisfactory results, but the optimal culture medium is yet to be developed for felids. In this study, the supplementation of the medium with insulin-like growth factors (IGF-I, IGF-II) and granulocyte-macrophage colony-stimulating factor (GM-CSF) coordinated with other items related to in vitro procedures, such as type of fertilisation and type of culture, are analysed. Finally, the addition of IGF-I and GM-CSF to the culture medium improved embryo production in a single-culture system. The supplementation with IGF-I increased the maturation rate of feline oocytes, as well as the morula and blastocyst rates of embryos, independently of the fertilisation method used. As only a small number of oocytes can be retrieved from wild species, this conclusion may be of special relevance for protocols for endangered felids.

**Abstract:**

Embryo production is a routine procedure in several species. However, in felids, the effectiveness of this approach is far behind that in the majority of laboratory species. The development of a suitable environment starts with the proper composition of culture media. Therefore, for the improvement of assisted reproduction techniques and their outcome in cats, this is an urgent task. As the addition of insulin-like growth factors (IGF-I, IGF-II) or granulocyte-macrophage colony-stimulating factor (GM-CSF) was beneficial in other mammalian species, this study aims to check whether these components, combined with other factors (such as type of fertilisation or type of culture) can provide a benefit in the felid culture system in current use. Thus, these supplements, in different concentrations and combinations, were merged with the use of two fertilisation techniques and randomly assigned to single or group culturing. The results showed that the addition of IGF-I and/or GM-CSF produced an increase in morula and blastocyst rate in a single culture system. In particular, the supplementation with 20 ng/mL of IGF-I incremented the maturation rate by 10% and significantly increased the morula and blastocyst rates in single culturing. This result is especially remarkable for wild felids, where only a few oocytes and/or embryos are available.

## 1. Introduction

As listed in the Red List of the International Union for Conservation of Nature (IUCN), 31 of the 38 Felidae species are suffering a decrease in their global population [1]. Nowadays, the remaining groups comprise only a few individuals and limited genetic resources need to be preserved for conservation breeding programs. Assisted reproductive techniques are used to guarantee the healthy genetic variability of new individuals by bringing together distant populations. It is basic for the success in this goal, as well as for contribution to the biological knowledge of these species, to handle consistent methods that can produce as many embryos of good quality as possible. Thus, optimisation of the embryo production system is urgent.

In vitro embryo production is a large-scale routine method in several species. In the case of the bovine industry, meeting the requirement of the dairy business, the number of in vitro produced embryos has already exceeded the number of those developed in vivo [2]. On the other hand, genome modification studies are based on in vitro mouse embryo production [3]. Currently, the fertilisation success in Bovinae, as well as in mouse embryos, reaches 85% [4,5]. The achievement of these rates is clearly due to the efforts in understanding the gamete and embryo requirements to not only environment parameters but also components in culture medium [6].

In felid species, where the domestic cat, *Felis catus*, is used as a laboratory model, the search for adequate conditions that would mimic the real scene has its own long history [7,8]. The fertilisation rate in the domestic cat remains about 60% [9]. Therefore, there is still room for improvement, and an optimal embryo culture medium for cats is yet to be developed.

The insulin-like growth factor (IGF) system includes, among surface receptors and binding proteins, insulin and two growth factors (IGF-I and IGF-II) [9]. The members of the IGF family are involved in a variety of roles promoting cell growth, proliferation, differentiation, and survival, among others, by acting as endocrine, paracrine and autocrine factors [10]. IGF-I is a small single-chain polypeptide consisting of 70 amino acids with a molecular weight of 7649 Da [11]. Stimulated by growth hormone to a greater extent, the production of IGF-I reaches its maximum in the liver, where its secretion to the bloodstream allows the endocrine function. Nevertheless, other tissues can also contribute to its production, determining in a broader context its autocrine/paracrine action [12,13].

As to female reproductive tissues, IGF-I is an indispensable element of the ovulatory pathway in the mouse [14]. IGF-IR has been found on bovine oocytes, cumulus cells, granulosa cells, and theca cells [15]. In cattle, the addition of IGF-I synergises with FSH in vitro to regulate aromatase activity in granulosa cells [16]. The addition of IGF-I to cumulus-oocyte complexes (COCs) enhances cumulus expansion and increases nuclear maturation in vitro [17]. Human preimplantation embryos express IGF-IR before the start of the production of IGF-I itself; hence, maternal IGF-I enhances their development [18]. Exogenous IGF-I also acts on embryos, improving their competence during the preimplantation period. It increases the blastocyst rate and total cell number in mice [19], and in cattle, it also decreases the rate of apoptosis [20,21]. In cats, all members of the IGF family are expressed differentially in the endometrium during pregnancy, suggesting that they act as regulatory factors for this condition [22]. IGF-I is a factor widely studied in the in vitro culture of cat follicles at different stages due to the relation with better results of follicular development, antrum formation, and proliferation of granulosa cells [23]. Few studies on the effect on early embryo development of IGF-II have been published; nevertheless, similar effects in mice were observed [24,25]. Recently it was reported that IGF-II improves developmental competence in oocytes from aged mice [26].

Granulocyte-macrophage colony-stimulating factor (GM-CSF) is a multifunctional cytokine produced in several tissues with different functions [27,28] when an adequate stimulus is present, whether hemopoietic or non-hemopoietic cell populations are concerned [29,30]. The multifocal production is related to several functions, not only the host’s responses to external stimuli and inflammatory and autoimmune conditions [27] but it is also involved in activating numerous pathways related to cell survival and proliferation and to functional activation [28]. In the reproductive tract of cattle and mice, among other species, this cytokine is secreted in both oviduct and endometrium [31,32] and synthesised by epithelial cells due to oestrogen stimulation. GM-CSF and its receptor were also described in human trophoblast cells [33,34]. When GM-CSF is added to an in vitro embryo culture, the rate of apoptosis decreases and cell proliferation is enhanced; therefore, blastocyst rates are higher in cattle [35] mice [36] and humans [37].

In this study, the effect of the addition of the factors IGF-I, IGF-II and GM-CSF during in vitro production of cat embryos was tested. To mimic the situation when few oocytes are obtained in non-domestic feline species, a single-embryo culture was compared to a group culture. Additionally, the influence of the fertilisation method was analysed, as ICSI is sometimes required in case of impaired sperm quality.

## 2. Materials and Methods

All chemicals were purchased from Sigma-Aldrich (Taufkirchen, Germany) unless stated otherwise.

### 2.1. Animals

Ovaries and testes were obtained from domestic cats neutered for population management reasons and kindly provided by local veterinary clinics from Novosibirsk or the animal shelter in Berlin. None of the animals was ovariectomized or castrated directly for this study. The age, breed, health status, or other individual parameters of the animals were not provided. The study was performed in three subsequent experiments during the breeding season of domestic cats. Specifically, Experiment A was performed in Novosibirsk (Russia) in January and February 2019. Experiment B was undertaken by the same researcher in Berlin (Germany) in March and April 2019, and Experiment C was performed in Berlin in May and June 2019.

### 2.2. Oocyte Retrieval and Sperm Collection

After surgery, gonads were placed into 50 mL conical Falcon tubes and transported in an expanded polystyrene box. Testes were transported without any medium, and ovaries, in Minimum Essential Medium Eagle HEPES modification (HEPES-MEM) supplemented with 1:100 (*v*:*v*) Antibiotic Antimycotic Solution and 3 mg/mL BSA. Ovaries were used immediately after arrival at the lab, while testes were in some cases kept at 4 °C overnight.

For oocyte retrieval, ovaries were sliced with a scalpel blade in washing medium (WM), consisting of M199 with 3 mg/mL bovine serum albumine, 1.4 mg/mL HEPES, 0.6 mg/mL sodium lactate, 0.25 mg/mL sodium pyruvate, 0.15 mg/mL L-glutamine, and 0.1 mg/mL cysteine. For Experiment A, penicillin/streptomycin (100 µg/mL) was used as the antibiotic, whereas gentamicin (0.055 mg/mL) was added in Experiment B and C. As previously described [38,39] follicles were dissected and COCs that looked like dark and homogenous ooplasm with several intact granulosa cell layers surrounding it were classified as high-quality and collected. The mean number of high-quality COCs obtained per ovary was similar to that in previous studies, ranging from three to four [40].

Sperm was used either fresh or frozen, depending on availability. For fresh preparation, vas deferens and cauda epididymis from cat testes were dissected and carefully minced in prewarmed M199 (1 mL) to enable the release of spermatozoa. The sample quality was evaluated for motility under a phase-contrast microscope at 20× magnification. The suspension was centrifuged at 300× *g* for 8 min, the supernatant was discarded, and a prewarmed medium was added to the pellet to adjust the concentration of motile sperm for fertilisation (see Section 2.4). Frozen sperm samples were thawed as previously detailed [41] by immersing the vial in a water bath at 38 °C for 90 s. The solution was then diluted 1:2 (*v*:*v*) with prewarmed M199. After 10 min of incubation at 38 °C, the sample quality was estimated. The sperm sample was washed from cryoprotectants by centrifuging at 300× *g* for 8 min and re-suspending in the fresh prewarmed medium until the desired concentration of motile sperm (see Section 2.4).

### 2.3. In Vitro Maturation (IVM)

The selected COCs were randomly distributed into different experimental groups. In Experiment A oocytes were subjected to group IVM, with 6–12 COCs per 500 µL of preincubated WM supplemented with eCG (1 IU/mL, Follimag, Mosagrogen, Russia) and hCG (5 IU/mL Chorulon, Intervet, The Netherlands). In Experiments B and C, a single IVM was performed by settling each oocyte in 20 µL of preincubated IVM medium composed of WM supplemented with hLH (0.2 IU/mL, L6420) and hFSH (0.5 IU/mL, Ferring, Kiel, Germany). Drops were covered with mineral oil and cultured at 38.5 °C in a humidified atmosphere with 5% CO_2_ for 24 h.

### 2.4. Fertilisation

Two fertilisation techniques were used. In Experiment A only in vitro fertilisation (IVF) was performed. For this, COCs were fertilised in groups of 6–12 in 500 µL of Ham’s-F10 medium supplemented with 5% FCS, 1 mM glutamine, 10 μg/mL heparin, and 50 μg/mL penicillin and streptomycin. Motile sperm cells were added to each group in the amount of 30.000 motile sperm cells per oocyte for 24 h. In Experiment B, IVF and intracytoplasmic sperm injection (ICSI) were performed in parallel. For IVF oocytes were cultured singly. Each COC was settled in 20 µL of WM supplemented with 2.2 IU/mL of heparin and cocultured with 20.000 motile sperm cells per drop for 18 h. In both cases, fertilisation was performed at 38.5 °C and 5% CO_2_ in a humidified atmosphere with 5% CO_2_.

In Experiments B and C, ICSI was used (see Section 2.6). COCs were therefore denuded by gently pipetting with a micropipette (The Stripper^®^, BioTipp, Waterford, Ireland). Denuded oocytes were examined for the extrusion of the polar body as a sign of metaphase II at 200× magnification under an inverted microscope (Axiovert 100, Carl Zeiss, Jena, Germany). Then, the matured oocytes were placed singly in 5-μL drops of WM supplemented with 0.5 mg/mL HEPES and sperm solution in 10 µL drops of polyvinylpyrrolidone (PVP, Origio, Berlin, Germany) diluted 1:2 in WM in a 6-cm petri dish covered with mineral oil. A sperm cell with normal morphology was immobilised and injected into the ooplasm.

### 2.5. In Vitro Embryo Culture (IVC)

Presumptive zygotes were cultured either singly or in groups of three embryos in 20-µL drops of embryo culture medium, Ham’s F-10 supplemented with 0.075 mg/mL L-glutamine and 5% FCS. For Experiment A, penicillin/streptomycin (50 µg/mL) was added, whereas in Experiments B and C, gentamycin (60 µg/mL) was used as the antibiotic component. Embryo culturing was performed at 38.5 °C and 5% CO_2_ in a 6-cm petri dish covered with mineral oil. In Experiments B and C, the conditions were the same except for reduced oxygen (5%).

Embryos were tested at 24-h intervals. Embryos that did not meet developmental criteria were fixed for fluorescence analysis to confirm the achieved developmental stage. In Novosibirsk, stopped embryos were placed into Eppendorf tubes with formaldehyde for fixation, whereas in Berlin they were dried on a slide before fixation in ethanol. Embryo culturing was performed for 7 days after fertilisation for Experiment A or proceeded for 5–6 days in Experiments B and C, when morulae were frozen for other experiments.

### 2.6. Experimental Design

The first part of this study, Experiment A, was carried out in Novosibirsk, whereas Experiment B and Experiment C were performed in Berlin, mainly by the same researcher. If not specified, the same procedures were applied. Media compositions in the two locations differed with respect to the antibiotic supplementations and hormones added for IVM.

In Experiment A, IVM (a total of 420 COCs) took place in group culture as previously reported [42], and after fertilisation by IVF two different types of culture were tested: single and group, referred to three zygotes per group. For both groups, different supplementation was applied to IVC, and five treatments were tested: (1) control group, (2) the addition of IGF-I at the concentrations 10 ng/mL; (3) the same with 20 ng/mL IGF-I, (4) the addition of GM-CSF at 2 ng/mL alone, or (5) in combination with a low dose of IGF-I corresponding to 10 ng/mL. It was shown earlier that IGF-I at 25 or 50 ng/mL improved embryo development of cat embryos, but lesser (5 mg/mL) and (higher 100 ng/mL) doses were ineffective [43]. Doses 10 and 20 ng/mL were never tested on cat embryos. GM-CSF at 2ng/mL was proven to improve preimplantation embryo development in different mammalian species [44,45,46]. At this dose, GM-CSF was successfully used in clinical trials with patients who underwent IVF [47].

In Experiment B, the effect of the fertilisation method combined or not combined with supplementation was tested. On the grounds of the results obtained in Experiment A, supplementation was restricted to the addition of 20 ng/mL of IGF-I to reduce the number of replicates. In this case, a total of 241 oocytes were matured in vitro with the presence or absence of the growth factor and fertilised either by IVF or ICSI by dividing each batch of oocytes after IVM randomly.

In Experiment C, the addition of a new factor, IGF-II, was tested in combination with IGF-I in comparison with the use of this growth factor alone added to single IVM and embryo culture of a total of 70 oocytes. The factor was applied at the concentration of 20 ng/mL in both cases. The IGF-II concentration was chosen according to the serum level in mice [26].

### 2.7. Statistics

Statistical analysis was performed with InStat^®^ (InStat, GraphPad Software, San Diego, CA, USA). Numbers of in vitro matured oocytes, cleaved oocytes, and embryos as well as cleavage, morula and blastocyst rates, were analysed using one-tailed exact probability from Fisher’s Exact Test. Differences were considered significant at *p* < 0.05.

## 3. Results

### 3.1. Experiment A

A total of 420 COCs were used in Experiment A (Novosibirsk). Groups contained between 31 and 81 oocytes per treatment as presented in Table 1. All the oocytes were matured and fertilised in vitro by the IVF technique. Zygotes were randomly assigned to one of the five different treatment groups for either single or group culture. Of 243 embryos, 102 were destined to single culture and 141 to group culture. The mean embryo cleavage rates were 59.7% in single cultures and 56.6% in group cultures, whether the media were supplemented or not. In the single-culture groups, when using the higher dose of IGF-I, significantly higher morula (87%) and blastocyst (43.5%) rates were achieved in comparison to the single-culture control (52.2% morulae, 13% blastocysts). The addition of low amounts of IGF-I and GM-CSF also resulted in a moderate but not significant increase in morulae (77.8% and 70.6%) and blastocysts (27.8% and 29.4%), whereas the combination of both factors brought about a significantly higher morula rate (81%) but no increase in the development to blastocysts as compared to the control. There were no significant differences between group cultures, although the comparison of the rates obtained with the same treatments showed some discrepancies. The morula and the blastocyst rates were affected by the type of embryo culture. Thus, the addition of 20 ng/mL of IGF-I produced a significantly higher blastocyst rate in single-culture embryos compared with the same treatment applied on group-culture embryos. Similarly, the morula rate obtained with the GM-CSF + IGF-I treatment was significantly lower when oocytes were cultured in groups.

### 3.2. Experiment B

In Experiment B (Berlin), 241 COCs were matured in vitro and fertilised by IVF, and 138, by ICSI. Approximately one-half of each group were cultured singly with or without the higher dose of IGF-I. For the ICSI group, in addition to cleavage and embryo development rates shown in Table 2, the maturation rate could be analysed by the presence of the polar body before sperm injection. Accordingly, the maturation rate in the control was 67.7% (69 of 102 COCs) vs. 77.5% (69 of 89 COCs) in the group where IGF-I was added. In the oocytes fertilised by ICSI, we observed that the cleavage rates in the control and treatment groups were the same; nevertheless, the morula rate tended to be higher in the IGF-I supplemented embryos. However, comparing IVF and ICSI, we observed that the cleavage rate did not reveal a significant difference between both fertilisation techniques. In the embryo development, we found a difference in the group not supplemented, where the morula rate was significantly higher in oocytes fertilised by IVF. The supplementation of embryos did not show significant differences between groups, but the cleavage rate in the group fertilised by IVF where IGF-I was added was slightly higher than in the group without the supplementation.

### 3.3. Experiment C

In Experiment C (Berlin), in order to increase the positive effect of IGF-I, IGF-II was added to observe whether the combination of both factors would exert a synergistic effect. As showed on Table 3, the use of IGF-I alone was compared with medium supplementation with a combination of IGF-I and IGF-II using single culturing and IVF. Nevertheless, the cleavage and morula rates remained relatively stable in both groups, being slightly higher in the group supplemented only with IGF-I. Of the 70 COCs used, about one-half cleaved and about 35% became morulae after five days of culturing.

## 4. Discussion

The efficiency of an in vitro embryo production method is measured by the number and quality of embryos that are produced routinely, with regard to their quality and the stage that they can achieve. Clearly, a deeper knowledge of the timing, environment and biological mechanisms involved in these procedures can help one to obtain much better results. Laboratory models are needed for wild species, as they allow us to develop the basis for techniques that later can be adapted to the special requirements of a target species if needed [48]. We already showed that techniques developed in the domestic cat can be successfully applied to wild cats [49,50,51]. Samples from wild cats are extremely rare and cannot be used for basic research. However, maximizing the use of these rare samples is important for conservation purposes. Current in vitro reproductive techniques for felids have a low success rate, as culture media are suboptimal. Therefore, developing and optimizing reliable protocols using domestic cats is a worthwhile investment. This study is focused on medium supplementation during IVM and embryo culture with different growth factors in relation to the type of culture or type of fertilisation. In Experiment A it is shown that cleavage rates are quite similar in general regardless of medium supplementation or whether we compare single and group culturing. The effect of growth factors was evident only when comparing later stages of embryo development. COCs are supposed to benefit from their neighbours by obtaining and secreting factors that have a positive impact on the maturation of all of them. In previous studies, singly matured oocytes showed a lower expression of IGF-IR when achieving the morula stage than group-cultured COCs [43], but the supplementation of culture medium with IGF-I enhanced the blastocyst rate up to the group culture level, meaning that IGF-I addition could increase the competence of embryos by replacing the benefits of group culturing and reducing the morula block.

In the present study also significantly higher morula and blastocyst rates were observed in the group of single-cultured COCs supplemented with 20 ng/mL of IGF-I as compared to single cultured control oocytes. In fact, the addition of the chosen factors generally enhanced embryo development in a single culture. In group culturing, the effect of IGF-I supplementation was less pronounced or even non-detectable, although the morula rate was slightly higher than in the control group. Taken together, our data from all three experiments confirm the positive effect of IGF-I on early embryo development.

In addition to IGF-I, GM-CSF was found to be a potential factor stimulating embryo development, as a significantly higher morula rate was obtained, but this effect was not consistent throughout the blastocyst stage, maybe due to the lower concentration of IGF-I added. We suppose that IGF-I addition to culture medium is a helpful component in overcoming deficiencies in culture, as under single-culture conditions, but other compounds are also needed to improve embryo production in the feline system. Nevertheless, overcoming the single culture drawbacks is a major step when working with exotic species. These studies could benefit from the addition of such components as only a few oocytes are usually available for maturation and embryo production, which makes single embryo cultures more desirable than group embryo culturing [50].

It was shown that GM-CSF does not rescue poor-quality embryos in humans, which means that even though the development of embryos treated with this factor tends to improve, the results are not statistically significant [52]. The outcome of single GM-CSF addition in Experiment A supports this statement, increasing the morula and blastocyst rates in single cultures when compared with the respective controls, but this trend was not exhibited under group culture conditions. In the future, it would be interesting to analyse the effect of GM-CSF addition to frozen/thawed embryos, as it was shown that this procedure increases blastocyst formation in humans more than twofold [37].

The effect of the growth factors did not vary with the different fertilisation methods used, as shown in Experiment B. Previous studies pointed out that the fertilisation method did not affect the outcome in cats [38]. Our study corroborates this observation, also demonstrating that, regardless of the technique used, the addition of growth factors produces no significant differences in embryo development. Before fertilisation, oocytes were examined for the presence of the polar body in the ICSI group, where the maturation rate showed a 10% increase in COCs treated with IGF-I. Although we do not have data on the maturation rate for the IVF group studied herein, the same effect can be conjectured, as reflected in a higher cleavage rate in the supplemented group. Supplementation of the maturation medium with IGF-I increased the in vitro maturation rate of oocytes recovered from ovaries at the follicular stage significantly, while it showed no difference in oocytes coming from the luteal stage [53]. However, a significantly higher maturation rate in high-quality oocytes supplemented with IGF-I was observed [54]; nevertheless, in low-quality COCs, there was no difference in maturation rate between the control and treatment groups. As for this study, only high-quality oocytes were chosen, this result supports the fact that here also a higher maturation rate in the group supplemented with IGF-I is obtained, indicating that the addition of IGF-I promotes maturation in cats.

The physiological effects of IGF-II, the third member of the IGF family, are less known. IGF-II is a single-chain peptide formed by 67 amino acids, and it shows about 70% structural similarity to IGF-I [11]. In mice, the presence of M6P/IGF-IIR starts at the two-cell stage of embryos, and the addition of external IGF-II increases the formation of blastocysts, as well as their cell number, supporting the role of this growth factor in early development [24,25]. To our knowledge, this factor has not been studied in vitro embryo cultures of cats. In Experiment C, the addition of this growth factor to the in vitro culture media of the embryos did not produce any benefit as compared with the use of IGF-I alone. Moreover, slightly higher rates were obtained when only IGF-I was added. Even though IGF-II binds to IGF-IR with lesser affinity [13], we hypothesize that the positive effect of IGF-I is reduced when IGF-II competes for IGF-I-specific binding sites.

## 5. Conclusions

Work with endangered species attaches the importance of sharing knowledge over borders and transferring techniques to the places where these species are still living in nature. The experiments reported here were conducted collaboratively by two institutions in Germany and Russia. Endangered felids, in particular, the Amur Tiger (*Panthera tigris tigris*), Amur Leopard *(Panthera pardus orientalis)*, and Snow Leopard (*Panthera uncia*), still live in the wild in Russia. Gaining basic knowledge on reproductive physiology and early embryo development in felids with the use of the domestic cat as model species is a prerequisite of the subsequent application of ART to endangered felid species conservation. This study covers various aspects of the action of IGF-I, IGF-II, and GM-CSF in the IVM of feline oocytes and culturing of cat embryos. We conclude that neither GM-CSF nor IGF-II bring about a significant increase in the desired outcome when added to the culture medium one by one. IGF-I, on the other hand, seems to have a positive effect on the maturation rate of single-cultured cat COCs. In single-cultured embryos, this effect also manifests itself as significantly higher morula and blastocyst rates. Single culturing is used in endangered species, as only a few oocytes and embryos can be obtained, and also in special cases where oocytes need to be tracked singly. Nevertheless, future studies are demanded to cover more factors in order to improve the felid embryo culture system up to the level comparable with other species.

## Figures and Tables

**Table 1:** Experiment A (Novosibirsk): Embryo development with different medium supplementation in single or group cultures.

Type of Culture	Treatment	N COCs	Embryo Development		
			N Cleaved Embryos (%)	N Morulae (%)	N Blastocysts (%)
Single Culture	Control	32	23 (71.9)	12 (52.2 ^a,b^)	3 (13.0 ^d^)
	IGF-I, 10 ng/mL	31	18 (58.1)	14 (77.8)	5 (27.8)
	IGF-I, 20 ng/mL	40	23 (57.5)	20 (87.0 ^a^)	10 (43.5 ^d,e^)
	GM-CSF, 2 ng/mL	33	17 (51.5)	12 (70.6)	5 (29.4)
	GM-CSF, 2ng/mL + IGF-I, 10 ng/mL	35	21 (60.0)	17 (81.0 ^b,c^)	3 (14.3)
Group Culture	Control	66	46 (69.7)	27 (58.7)	5 (10.9)
	IGF-I, 10 ng/mL	33	21 (63.6)	15 (71.4)	2 (9.5)
	IGF-I, 20 ng/mL	36	21 (58.3)	13 (61.9)	1 (4.8 ^e^)
	GM-CSF, 2 ng/mL	33	16 (48.5)	8 (50.0)	1 (6.3)
	GM-CSF, 2ng/mL + IGF-I, 10 ng/mL	81	37 (45.7)	19 (51.4 ^c^)	4 (10.8)

^a,b,c,d,e^ Within a column, the same superscripts indicate significant differences.

**Table 2:** Experiment B (Berlin): Summary of the development of embryos obtained with IVM and different fertilisation methods with or without the addition of 20 ng/mL of IGF-I to single cultures.

Fertilisation Technique	IGF-I −				IGF-I +			
	N COCs	N Mature Oocytes (%)	N Cleaved Embryos (%) ^#^	N Morulae (%)	N COCs	N Mature Oocytes (%)	N Cleaved Embryos (%)	N Morulae (%)
IVF	118	n.a.	46 (38.9)	23 (50.0 ^a^)	123	n.a.	58 (47.2)	20 (34.5)
ICSI	102	69 (67.7)	29 (28.4)	6 (20.7 ^a^)	89	69 (77.5)	29 (32.6)	8 (27.6)
IVF + ICSI	220	n.a.	75 (34.1)	29 (38.7)	212	n.a.	87 (41.0)	28 (32.2)

^#^ Cleavage rate is calculated from the total number of COCs. ^a^ Within a column, the same superscripts indicate significant differences. n.a.: not applicable.

**Table 3:** Experiment C (Berlin): Effects of the addition of 20 ng/mL of IGF-I vs. the supplementation with 20 ng/mL of each of IGF-I and IGF-II on single IVM and culture embryo development after IVF.

Treatment	N COCs	N Cleaved Embryos (%)	N Morulae (%)
IGF-I	34	19 (55.9)	7 (36.8)
IGF-I + IGF-II	36	18 (50.0)	6 (33.3)

No significant differences between treatments were found.

## Data Availability

The data presented in this study are available on request from the corresponding author.

## References

[1] IUCN Iucn 2020. The Iucn Red List of Threatened Species. Version 2020-3. http://www.Iucnredlist.org.

[2] Viana J. (2018). 2017 statistics of embryo production and transfer in domestic farm animals. Embryo Technol. Newsl..

[3] Fujii W., Kawasaki K., Sugiura K., Naito K. (2013). Efficient generation of large-scale genome-modified mice using grna and cas9 endonuclease. Nucleic AIDS Res..

[4] Ermisch A.F., Herrick J.R., Pasquariello R., Dyer M.C., Lyons S.M., Broeckling C.D., Rajput S.K., Schoolcraft W.B., Krisher R.L. (2020). A novel culture medium with reduced nutrient concentrations supports the development and viability of mouse embryos. Sci. Rep..

[5] Ferré L.B., Kjelland M.E., Strøbech L.B., Hyttel P., Mermillod P., Ross P.J. (2020). Review: Recent advances in bovine in vitro embryo production: Reproductive biotechnology history and methods. Animal.

[6] Hansen P.J. (2006). Realizing the promise of ivf in cattle—An overview. Theriogenology.

[7] Luvoni G.C., Colombo M., Morselli M.G. (2018). The never-ending search of an ideal culture system for domestic cat oocytes and embryos. Reprod. Domest. Anim..

[8] Herrick J.R., Bond J.B., Magarey G.M., Bateman H.L., Krisher R.L., Dunford S.A., Swanson W.F. (2007). Toward a feline-optimized culture medium: Impact of ions, carbohydrates, essential amino acids, vitamins, and serum on development and metabolism of in vitro fertilization-derived feline embryos relative to embryos grown in vivo. Biol. Reprod..

[9] Cohick W.S., Clemmons D.R. (1993). The insulin-like growth factors. Annu. Rev. Physiol..

[10] Allard J.B., Duan C. (2018). Igf-binding proteins: Why do they exist and why are there so many?. Front. Endocrinol..

[11] Rinderknecht E., Humbel R.E. (1978). The amino acid sequence of human insulin-like growth factor i and its structural homology with proinsulin. J. Biol. Chem..

[12] Laron Z. (2001). Insulin-like growth factor 1 (igf-1): A growth hormone. Mol. Pathol..

[13] Le Roith D. (2003). The insulin-like growth factor system. Exp. Diabesity Res..

[14] Baker J., Hardy M.P., Zhou J., Bondy C., Lupu F., Bellvé A.R., Efstratiadis A. (1996). Effects of an igf1 gene null mutation on mouse reproduction. Mol. Endocrinol..

[15] Nuttinck F., Charpigny G., Mermillod P., Loosfelt H., Meduri G., Freret S., Grimard B., Heyman Y. (2004). Expression of components of the insulin-like growth factor system and gonadotropin receptors in bovine cumulus–oocyte complexes during oocyte maturation. Domest. Anim. Endocrinol..

[16] Spicer L.J., Chamberlain C.S., Maciel S.M. (2002). Influence of gonadotropins on insulin- and insulin-like growth factor-i (igf-i)-induced steroid production by bovine granulosa cells. Domest. Anim. Endocrinol..

[17] Lorenzo P., Illera M., Illera J., Illera M. (1994). Enhancement of cumulus expansion and nuclear maturation during bovine oocyte maturation in vitro by the addition of epidermal growth factor and insulin-like growth factor i. J. Reprod. Fertil..

[18] Lighten A.D., Moore G.E., Winston R.M., Hardy K. (1998). Routine addition of human insulin-like growth factor-i ligand could benefit clinical in-vitro fertilization culture. Hum. Reprod..

[19] Lin T.C., Yen J.M., Gong K.B., Hsu T.T., Chen L.R. (2003). Igf-1/igfbp-1 increases blastocyst formation and total blastocyst cell number in mouse embryo culture and facilitates the establishment of a stem-cell line. BMC Cell Biol..

[20] Velazquez M., Korsawe K., Niemann H. (2008). The effects of physiological and non-physiological insulin-like growth factor-1 (igf-1) concentrations on the in vitro development of bovine embryos. Reprod. Domest. Anim..

[21] Makarevich A., Markkula M. (2002). Apoptosis and cell proliferation potential of bovine embryos stimulated with insulin-like growth factor i during in vitro maturation and culture. Biol. Reprod..

[22] Korkmaz Ağaoğlu Ö., Ağaoğlu A.R., Özmen Ö., Saatci M., Schäfer-Somi S., Aslan S. (2020). Expression of the insulin-like growth factor (igf) gene family in feline uterus during pregnancy. Biotech. Histochem..

[23] Alves E.A., Padilha L., Savi P.A., Apparicio M.F., Mostachio G.Q., Motheo T.F., Pires-Buttler E.A., Vicente W.R., Luvoni G.C. (2012). In vitro survival of follicles collected from domestic cats’ ovaries at different stages of oestrous cycle and cultured with igf-1. Reprod. Domest. Anim..

[24] Harvey M.B., Kaye P.L. (1992). Igf-2 stimulates growth and metabolism of early mouse embryos. Mech. Dev..

[25] Rappolee D.A., Sturm K.S., Behrendtsen O., Schultz G.A., Pedersen R.A., Werb Z. (1992). Insulin-like growth factor ii acts through an endogenous growth pathway regulated by imprinting in early mouse embryos. Genes Dev..

[26] Muhammad T., Wan Y., Sha Q., Wang J., Huang T., Cao Y., Li M., Yu X., Yin Y., Chan W.Y. (2020). Igf2 improves the developmental competency and meiotic structure of oocytes from aged mice. Aging.

[27] Burgess A.W., Metcalf D. (1980). The nature and action of granulocyte-macrophage colony stimulating factors. Blood.

[28] Hercus T.R., Broughton S.E., Ekert P.G., Ramshaw H.S., Perugini M., Grimbaldeston M., Woodcock J.M., Thomas D., Pitson S., Hughes T. (2012). *;* et al. The gm-csf receptor family: Mechanism of activation and implications for disease. Growth Factors.

[29] Hamilton J.A. (2019). Gm-csf in inflammation. J. Exp. Med..

[30] Metcalf D. (1981). Hemopoietic colony stimulating factors. Tissue Growth Factors.

[31] De Moraes A.A.S., Paula-Lopes F.F., Chegini N., Hansen P.J. (1999). Localization of granulocyte-macrophage colony-stimulating factor in the bovine reproductive tract. J. Reprod. Immunol..

[32] Robertson S.A., Mayrhofer G., Seamark R.F. (1992). Uterine epithelial cells synthesize granulocyte-macrophage colony-stimulating factor and interleukin-6 in pregnant and nonpregnant mice. Biol. Reprod..

[33] Jokhi P., King A., Loke Y. (1994). Immunology: Production of granulocyte-macrophage colony-stimulating factor by human trophoblast cells and by decidual large granular lymphocytes. Hum. Reprod..

[34] Jokhi P., King A., Jubinsky P.T., Loke Y. (1994). Demonstration of the low affinity α subunit of the granulocyte-macrophage colony-stimulating factor receptor (gm-csf-rα) on human trophoblast and uterine cells. J. Reprod. Immunol..

[35] De Moraes A.A., Hansen P.J. (1997). Granulocyte-macrophage colony-stimulating factor promotes development of in vitro produced bovine embryos. Biol. Reprod..

[36] Robertson S.A., Sjöblom C., Jasper M.J., Norman R.J., Seamark R.F. (2001). Granulocyte-macrophage colony-stimulating factor promotes glucose transport and blastomere viability in murine preimplantation embryos. Biol. Reprod..

[37] Sjoblom C., Wikland M., Robertson S.A. (1999). Granulocyte-macrophage colony-stimulating factor promotes human blastocyst development in vitro. Hum. Reprod..

[38] Waurich R., Ringleb J., Braun B.C., Jewgenow K. (2010). Embryonic gene activation in in vitro produced embryos of the domestic cat (*Felis catus*). Reproduction.

[39] Jewgenow K., Fernandez-Gonzalez L., Jansch S., Viertel D., Zahmel J. (2019). Brilliant cresyl blue staining allows the selection for developmentally competent immature feline oocytes. Theriogenology.

[40] Hribal R., Jewgenow K., Braun B.C., Comizzoli P. (2013). Influence of culture medium composition on relative mrna abundances in domestic cat embryos. Reprod. Domest. Anim..

[41] Klaus C., Eder S., Franz C., Muller K. (2016). Successful cryopreservation of domestic cat (*Felis catus*) epididymal sperm after slow equilibration to 15 or 10 degrees c. Reprod. Domest. Anim..

[42] Sowinska N., Zahmel J., Nizanski W., Hribal R., Fernandez-Gonzalez L., Jewgenow K. (2020). Meiotic status does not affect the vitrification effectiveness of domestic cat oocytes. Animals.

[43] Thongkittidilok C., Tharasanit T., Sananmuang T., Buarpung S., Techakumphu M. (2014). Insulin-like growth factor-1 (igf-1) enhances developmental competence of cat embryos cultured singly by modulating the expression of its receptor (igf-1r) and reducing developmental block. Growth Horm. IGF Res. Off. J. Growth Horm. Res. Soc. Int. IGF Res. Soc..

[44] Brusentsev E.Y., Abramova T.O., Rozhkova I.N., Igonina T.N., Naprimerov V.A., Feoktistova N.Y., Amstislavsky S.Y. (2015). Cryopreservation and in vitro culture of preimplantation embryos in djungarian hamster (*Phodopus sungorus*). Reprod. Domest. Anim..

[45] Amstislavsky S., Brusentsev E., Kizilova E., Igonina T., Abramova T., Rozhkova I. (2015). Embryo cryopreservation and in vitro culture of preimplantation embryos in campbell’s hamster (*Phodopus campbelli*). Theriogenology.

[46] Desai N., Kattal N., Abdel Hafez F.F., Szeptycki-Lawson J., Goldfarb J. (2007). Granulocyte-macrophage colony stimulating factor (gm-csf) and co-culture can affect post-thaw development and apoptosis in cryopreserved embryos. J. Assist. Reprod. Genet..

[47] Armstrong S., MacKenzie J., Woodward B., Pacey A., Farquhar C. (2020). Gm-csf (granulocyte macrophage colony-stimulating factor) supplementation in culture media for women undergoing assisted reproduction. Cochrane Database Syst. Rev..

[48] Amstislavsky S., Mokrousova V., Brusentsev E., Okotrub K., Comizzoli P. (2019). Influence of cellular lipids on cryopreservation of mammalian oocytes and preimplantation embryos: A review. Biopreserv. Biobank.

[49] Fernandez-Gonzalez L., Hribal R., Stagegaard J., Zahmel J., Jewgenow K. (2015). Production of lion (*Panthera leo*) blastocysts after in vitro maturation of oocytes and intracytoplasmic sperm injection. Theriogenology.

[50] Zahmel J., Fernandez-Gonzalez L., Jewgenow K., Müller K. (2019). Felid-gamete-rescue within eaza-efforts and results in biobanking felid oocytes and sperm. J. Zoo Aquar. Res..

[51] Amstislavsky S., Brusentsev E., Kizilova E., Mokrousova V., Kozhevnikova V., Abramova T., Rozhkova I., Naidenko S. (2018). Sperm cryopreservation in the far-eastern wildcat (*Prionailurus bengalensis euptilurus*). Reprod. Domest. Anim..

[52] Rodriguez-Wallberg K.A., Munding B., Ziebe S., Robertson S.A. (2020). Gm-csf does not rescue poor-quality embryos: Secondary analysis of a randomized controlled trial. Arch. Gynecol. Obstet..

[53] Yıldırım K., Vural M.R., Küplülü S., Ozcan Z., Polat I.M. (2014). The effects of egf and igf-1 on fsh-mediated in vitro maturation of domestic cat oocytes derived from follicular and luteal stages. Reprod. Biol..

[54] Kitiyanant Y., Saikhun J., Pavasuthipaisit K. (2003). Somatic cell nuclear transfer in domestic cat oocytes treated with igf-i for in vitro maturation. Theriogenology.

